# Case report: Detection of non-O1/non-O139 *Vibrio cholerae* in a patient with hepatic space-occupying lesions using metagenomic next-generation sequencing

**DOI:** 10.3389/fmed.2024.1483027

**Published:** 2024-11-19

**Authors:** Wei Zhang, Li Xiao, Xingxing Shan, Bing Dai, Chunyan Tang, Jianchun Xian, Yan Yu

**Affiliations:** ^1^Changsha KingMed Diagnostics Group Co., Ltd., Changsha, Hunan, China; ^2^Department of Infection and Hepatology, Taizhou People's Hospital Affiliated to Nanjing Medical University, Taizhou, China; ^3^Nanjing KingMed Diagnostics Group Co., Ltd., Nanjing, Jiangsu, China

**Keywords:** non-O1/non-O139 *Vibrio cholerae*, hepatic space-occupying lesions, culture, metagenomic next-generation sequencing, polymerase chain reaction

## Abstract

**Introduction/background:**

*Vibrio cholerae* is the causative agent of the human intestinal infectious disease cholera, which includes a variety of serogroups. However, there have been very few cases of hepatic space-occupying lesions associated with this infection. Currently, there are various methods for detecting this pathogen, including metagenomic sequencing, which enables quicker and more accurate identification. In this study, metagenomic sequencing is employed to accurately identify non-O1/O139 *Vibrio cholerae* infections by analyzing the genetic material present in clinical samples.

**Presentation of case:**

A 75-year-old man presented with diarrhea and fever after consuming crabs. The initial treatment improved the diarrhea, but a liver abscess developed later. Magnetic resonance imaging (MRI) of the liver revealed a hepatic space-occupying lesion. Upon further investigation, a Gram-negative, rod-shaped bacterium was cultured from the patient’s liver puncture fluid, and *Vibrio cholerae* was detected in the same fluid using metagenomic next-generation sequencing (mNGS). The pathogen was confirmed to be non-O1/non-O139 *Vibrio cholerae* (*NOVC*) using polymerase chain reaction (PCR). Following treatment with piperacillin/tazobactam sodium and moxifloxacin, the patient’s body temperature returned to normal, the liver abscess improved significantly, and he was subsequently discharged from the hospital.

**Discussion:**

This case study describes an elderly male patient with a hepatic space-occupying lesion. Multiple cultures of specimens failed to identify the underlying cause; however, advanced techniques such as mNGS and PCR confirmed an *NOVC* infection. This indicates that mNGS can serve as a valuable tool in diagnosing cases of unexplained liver infections.

**Conclusion:**

The use of mNGS is significant for detecting and clinically diagnosing infectious pathogens in patients with unexplained space-occupying lesions.

## 1 Introduction

*Vibrio cholerae* is a Gram-negative bacteria, belonging to the genus *Vibrio*. It is an aerobic, water-borne pathogen. Currently, there are more than 200 *Vibrio cholerae* serogroups (1). Among these, O1 and O139 isolates typically produce the cholera toxin (2). Infection after consuming water or food contaminated with *Vibrio cholerae* can lead to cholera, a severe intestinal infectious disease characterized by severe diarrhea and vomiting. If not treated in time, the mortality rate can reach up to 70%.

The non-O1/non-O139 *Vibrio cholerae* (*NOVC*) isolates cannot produce toxins and, therefore, cannot cause cholera. However, they can cause bacteremia and invasive extraintestinal diseases (3, 4). The most common clinical manifestations of *NOVC* infection include gastroenteritis, but they can also cause septicemia (5), chronic otitis media (6), oral infections (7), necrotizing fasciitis (8), endophthalmitis (9), meningitis (10), and other invasive infections. There have been only a few reports of this bacterium causing hepatic space-occupying lesions.

Metagenomic next-generation sequencing (mNGS) is a culture-free and bias-free pathogen detection technology based on next-generation sequencing technology. It can simultaneously detect a variety of pathogens including bacteria, fungi, viruses, and parasites using high-throughput sequencing of DNA and/or RNA directly extracted from clinical samples, followed by data comparison and bioinformatics analysis (11, 12). Currently, it is gradually being transferred from research to clinical laboratories and used to identify pathogens in different infected parts of the body, such as respiratory infections, central nervous system infections, bloodstream infections, gastrointestinal infections, ocular infections, urinary tract infections, and hepatobiliary infections (11–13). Studies have shown that mNGS detection can be used to diagnose unknown infections in body fluids. This study tested 182 body fluids from 160 patients with acute illness using two sequencing platforms for mNGS. The results were compared to other diagnostic methods, such as culture, 16S bacterial PCR, and/or 28S internal donated ribosomal gene spacer (28S ITS) functional PCR. The sensitivity and specificity of the test for bacteria and fungi were over 75%, as determined by Illumina sequencing (14).

## 2 Case report

A 75-year-old man was admitted with fever and diarrhea. Routine examination and inquiry revealed that the patient had a clear history of seafood consumption before the onset of the disease. The patient consumed crabs that had been stored in the refrigerator for 8 days before developing symptoms of diarrhea and fever. Levofloxacin was used for anti-infection treatment in a local hospital, and the symptoms of diarrhea were relieved, but liver abscesses appeared. Later, the patient was transferred to our hospital for treatment. After undergoing an MRI examination of the liver at our hospital, a hepatic space-occupying lesion was identified.

**Figure 1:**
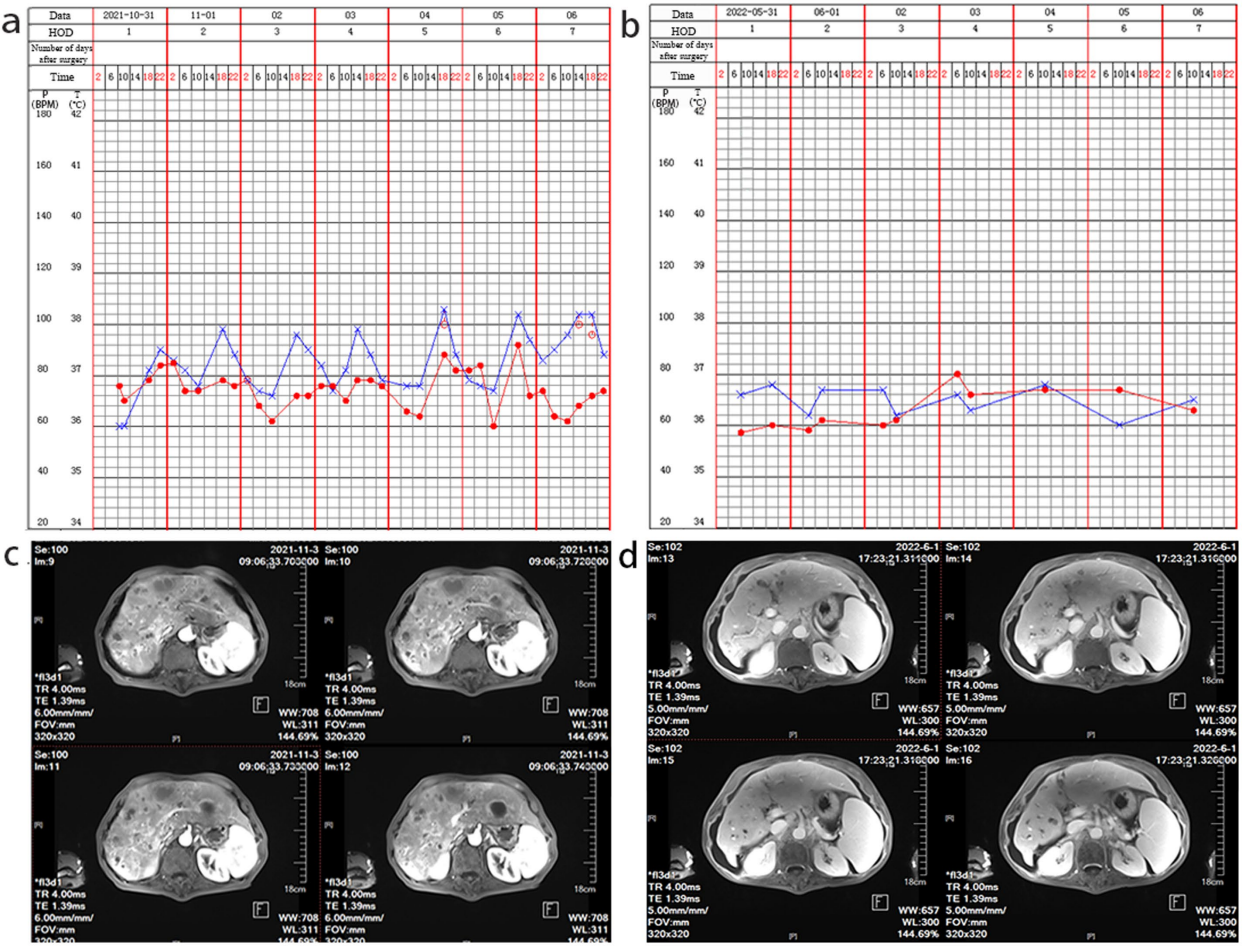
Nursing record sheet and liver MRI examination. (a) At the time of admission, a low fever was observed. (b) A follow-up after treatment indicated that the admission examination reveals a normal body temperature. (c) The initial liver MRI examination shows hepatic space-occupying lesions; (d) After treatment, a follow-up liver MRI examination shows improvement in liver abscess.

On admission, his vital signs were as follows: white blood cell (WBC) count was 10.03 × 10^9/L, with 69.3% neutrophils, 21.5% lymphocytes, and 7.0% monocytes. The concentration of procalcitonin (PCT) was 0.120 ng/mL, and the serum C-reactive protein concentration was 81.76 mg/L. Additionally, the patient had a mild fever upon admission (Figure 1a). Furthermore, the liver MRI scan revealed a hepatic space-occupying lesion (Figure 1c). No white blood cells were found in the smear of the liver puncture fluid.

Based on the MRI results, a liver abscess was suspected 1 week after admission, and a 21-day regimen of piperacillin/tazobactam sodium (4.5 g every 8 h) and moxifloxacin hydrochloride (0.4 g once daily) was started to treat the infection. Although there was a reduction in the size of the lesion, it did not completely disappear. Then, the patient’s condition was reassessed. Blood, feces, and liver puncture fluid samples were collected. After disinfecting the skin, approximately 5 mL of blood was collected through venipuncture. For the stool sample, a swab was used to collect an amount equivalent to the size of a peanut (approximately 5 g).

**Figure 2:**
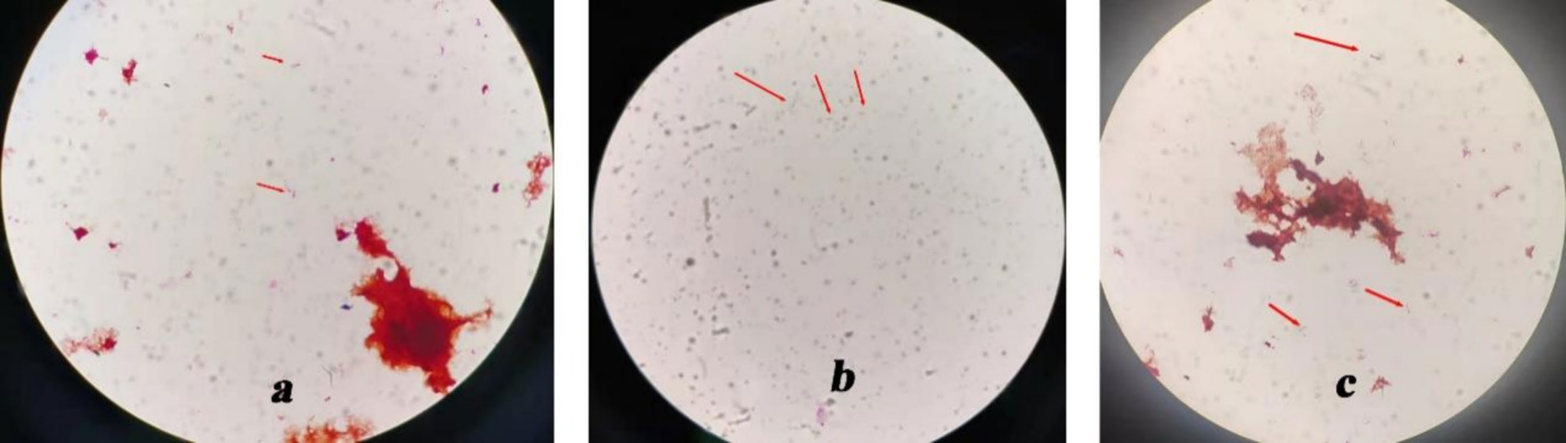
Culture results show a Gram-negative curved rod-shaped bacterium. (×1,000). (a) Gram-negative curved rod-shaped bacterium in blood agar. (b) Gram-negative curved rod-shaped bacterium in BacT/ALERT® FA culture bottles. (c) Gram-negative curved rod-shaped bacterium in a blood plate, isolated from BacT/ALERT® FA culture bottles.

In addition, under the guidance of CT or ultrasound, an 8-ml sample of the liver abscess fluid was aspirated through a puncture. All of the specimens were preserved and cultured at Nanjing KingMed Diagnostics (a third-party laboratory). Blood culture results were negative. *Escherichia coli* and *Enterococcus faecium* were identified in stool culture. The liver puncture fluid was inoculated onto a Columbia blood plate. After 24 h of incubation, a small amount of curved, Gram-negative, rod-shaped bacterium was observed growing on the blood agar under the microscope (Figure 2a).

The liver puncture fluid was also inoculated into a BacT/ALERT® FA culture bottle. After 120 h of incubation, the culture medium was taken from the BacT/ALERT® FA culture bottle, and Gram-negative rod-shaped bacteria with slight swelling and curvature were observed under a microscope (Figure 2b). The colony morphology suggested the presence of a rod-shaped bacterium. Subsequently, the liquid in the bottle was transferred to Columbia blood plates for further cultivation. After 24 h, curved Gram-negative rod-shaped bacteria were observed under the microscope (Figure 2c). Unfortunately, pathogen identification was not possible.

**Figure 3:**
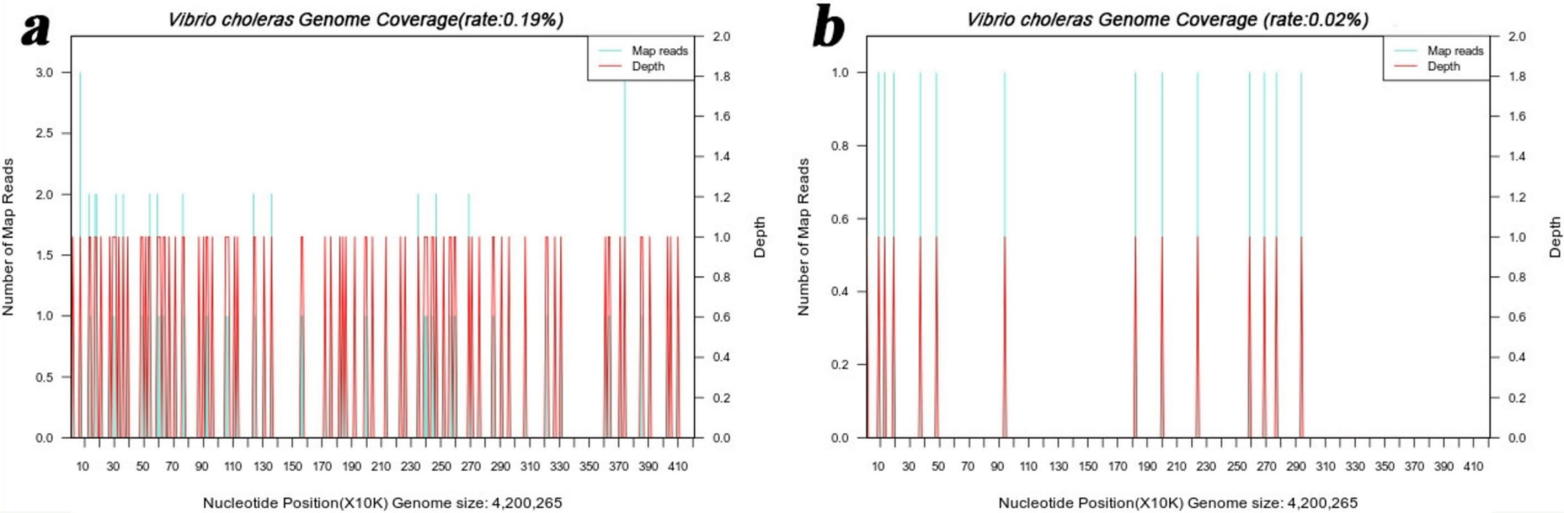
mNGS results of the liver puncture fluid. (a) The DNA mNGS results show that the coverage of *Vibrio cholerae* was 0.19%. (b) The RNA mNGS results show that the coverage of *Vibrio cholerae* was 0.02%.

**Figure 4:**
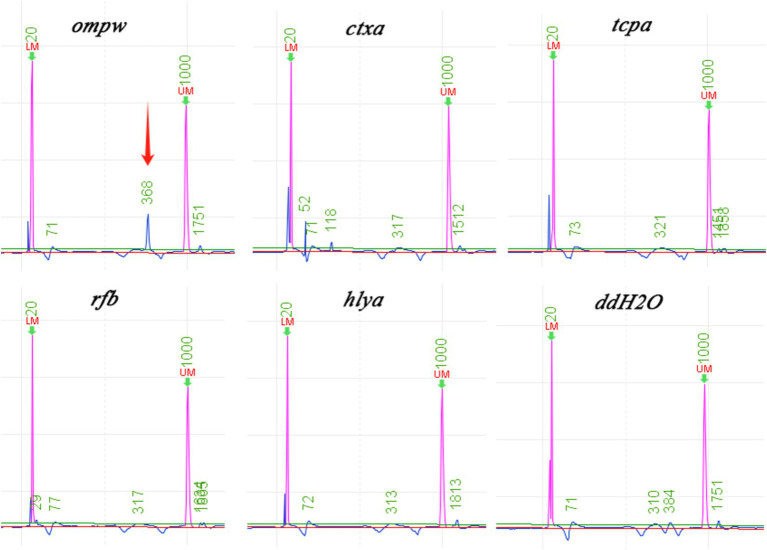
Results of the gene PCR. Except for *ompw*, other O1/O139 *Vibrio cholerae* toxicity-related genes are not amplified by PCR. (*ompw-positive*, *ctxa-negative*, *tcpa-negative*, *rfb-negative*, and *hlya-negative*).

To further confirm the pathogen of infection, mNGS detection was conducted. Subsequently, the liver puncture fluid was sent for mNGS testing (KingMed Diagnostics, Changsha, China). The experimental process of mNGS includes the extraction of nucleic acids (DNA and/or RNA), fragmentation, library preparation, and sequencing. By analyzing and processing the sequencing data, the microorganisms found in the specimen can be identified and studied. The mNGS results reported a *V. cholerae* infection. DNA mNGS detected 108 sequences that can be mapped to *V. cholerae*, with a coverage of 0.19% (Figure 3a). RNA mNGS detected 14 sequences that can be mapped to *V. cholerae*, with a coverage of 0.02% (Figure 3b). To verify the serotype of *V. cholerae*, five genes were amplified by PCR in the sliver puncture fluid, including *ompw*, *ctxa*, *tcpa*, *rfb*, and *hlya* (Table 1). However, *ctxa*, *tcpa*, *rfb*, and *hlya* did not amplify any specific fragments (Figure 4). Finally, the confirmation of the *NOVC* strain was achieved by mNGS and PCR at Changsha KingMed Diagnostics (a third-party laboratory).

**Table 1:** Primer for *Vibrio cholerae* verification.

Primer name	Sequence (5′ to 3′)	Base number	Gene description	References
*VCompw*-F	AGTCCTCGCTGCTACGCCATT	21	A gene of an outer membrane protein	(27, 28)
*VCompw*-R	CTGCTTTGTAGGTTGCCGTTGTTTC	25		
*VCctxa*-F	GACGGGATTTGTTAGGCACGATGAT	25	Cholera toxin gene	(29)
*VCctxa*-R	GCATGATGAATCCACGGCTCTTCC	24		
*VCtcpa*-F	TGGCTCAGCGTGCGATTGATTC	22	Toxin coregulated pilus A	(28)
*VCtcpa*-R	AGCACAAAGGTTCTGAACATGGGTA	25		
*VCrfb*-F	ACTGACGGATGGTGAAGTGATTGC	24	Antigen-specific gene of *Vibrio cholerae* O1 and O139	(30)
*VCrfb*-R	CTCTGTTGCTACTGCCGATTCTTGT	25		
*VChlya*-F	TTGTTAGAGGCACCACGCACTG	22	*Vibrio cholerae-specific* hemolysin genes	(29)
*VChlya*-R	TTCGGACCATCTCCACTGACTTCA	24		

This patient had a clear history of seafood consumption before the onset of the disease. Subsequently, it was confirmed to be *NOVC* through mNGS and PCR testing. Based on experience and recommendations from the ninth edition of “*Infectious Diseases*” by the People’s Health Publishing House, quinolone drugs are preferred for treating cholera infection. Therefore, the use of a moxifloxacin antibiotic was continued for treatment. After 40 days of treatment, the patient’s temperature tended to be stable, and he was discharged without fever. After 7 months of follow-up, the body temperature was normal (Figure 1b). After anti-infection treatment, the hepatic space-occupying lesions improved (Figure 1d).

## 3 Discussion

In this report, a 75-year-old man with *NOVC* infection was described. He suffered from hepatic space-occupying lesions and had consumed crabs that had been refrigerated for a long time. To the best of our knowledge, this is the first case of *NOVC* infection identified using mNGS in a patient with hepatic space-occupying lesions.

*Vibrio cholerae* is widely distributed in aquatic environments. Humans can contract an infection by consuming contaminated food, especially seafood and fish (15). Over the last 20 years, diseases caused by *NOVC* have increased steadily worldwide (15).

Studies have shown that infections caused by *NOVC* are more common in patients with underlying diseases or low immunity. Cirrhosis is the most common risk factor in mainland China, followed by malignancy, hematological malignancies, and diabetes (5, 16). *NOVC* bacteremia (17) and septicemia (18) have been found in patients with liver cirrhosis. However, *NOVC* sepsis was also reported in a patient who had no underlying disease (19). As stated in the case, the patient had liver space-occupying symptoms, but the early symptoms were insufficient to support a diagnosis of *NOVC* infection.

The patient’s WBC count was slightly higher, whereas the cell proportion and PCT were normal. In addition, C-reactive protein (CRP) levels increased significantly, and the patient’s body temperature increased, indicating an infection. After culturing samples from the blood, feces, and liver puncture fluid, no bacteria were detected. *Escherichia coli* and *Enterococcus faecium* were cultured from feces. Curved Gram-negative, rod-shaped bacteria were found in the liver puncture fluid, and the bacterial morphology was consistent with that of *V. cholerae*. However, the culture method is time-consuming and has limitations (20). Due to limited conditions, it was not possible to cultivate a single colony and conduct drug sensitivity tests. Afterward, *Vibrio cholerae* was detected using mNGS, and the toxicity-related genes of O1/O139 *Vibrio cholerae* were not amplified by PCR, confirming that the bacterium is *NOVC*. Following treatment based on clinical experience and mNGS results, the patient’s physical signs improved. *NOVC* was not found in blood and fecal cultures, demonstrating the importance of selecting the appropriate sample type. Due to the rarity of the *NOVC* infection, its diagnosis may be challenging. Agglutination with O1 antiserum can be used to exclude other types of *Vibrio cholerae* infection, which is typically performed in clinical laboratories and can be difficult for initial diagnosis (21).

However, mNGS is increasingly used to detect pathogens directly from clinical specimens (20). At present, mNGS is used to detect and diagnose secondary infection in patients with severe *Legionella pneumonia* after treatment (22). In addition, research has shown that mNGS has advantages in terms of speed and sensitivity for detecting pathogens in mixed lung infections (23). Now, mNGS can be used to identify pathogens in infectious CNS diseases, providing certain advantages over traditional detection methods (24). Rare pathogens can also be identified using mNGS, such as *Chlamydia psittaci*, *Orientia tsutsugamushi*, and others (25, 26). In this case, the liver puncture fluid was detected using mNGS technology, which confirmed the presence of *V. cholerae*. In general, this shows that the detection of unidentified infected body fluids through mNGS is of significant importance for clinical diagnosis.

## Data Availability

The original contributions presented in the study are included in the article/supplementary material, further inquiries can be directed to the corresponding author/s.
